# Supplementary figures and images for: Genome-Wide Analysis of Differentially Expressed mRNAs and lncRNAs in Koi Carp Infected with Koi Herpesvirus
Source: Viruses. 2022 Nov 18;14(11):2555. doi: 10.3390/v14112555 (PMC9694643; doi:10.3390/v14112555)

A

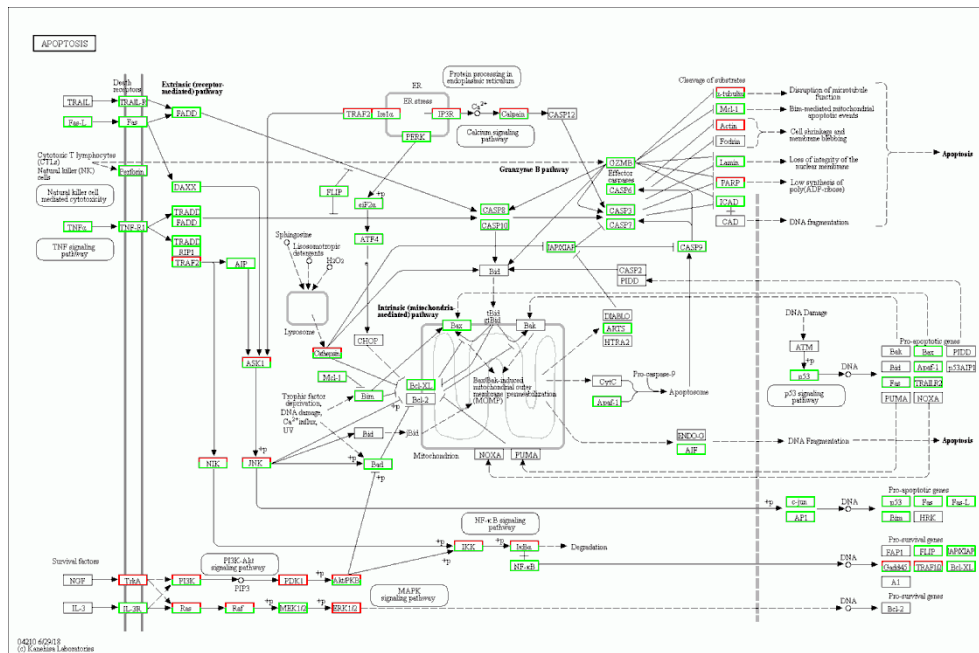**B**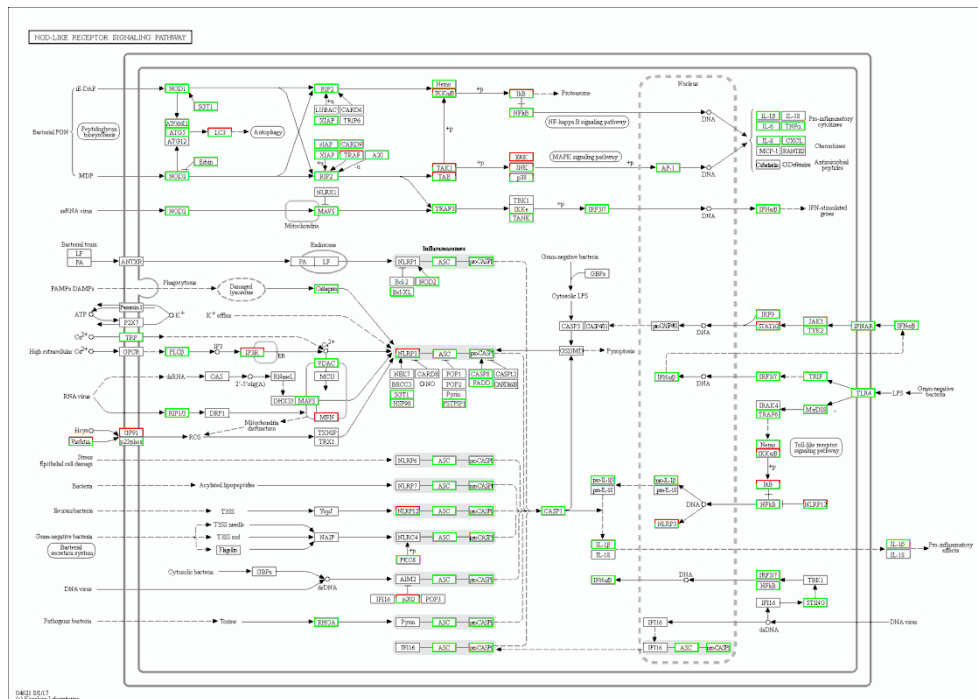

C

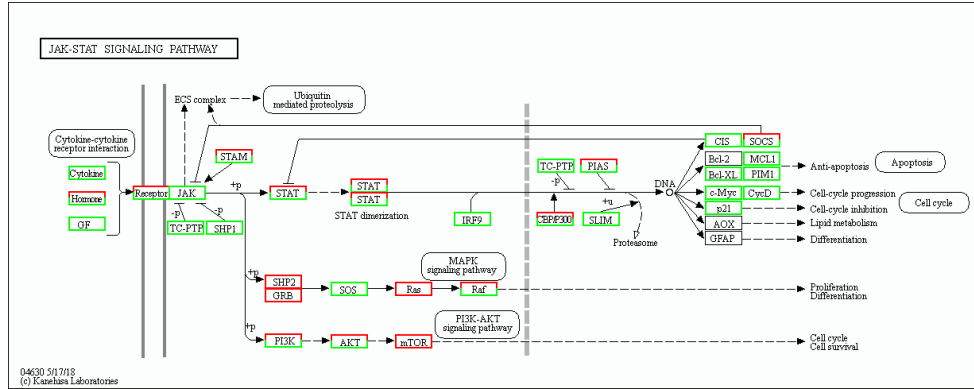

D

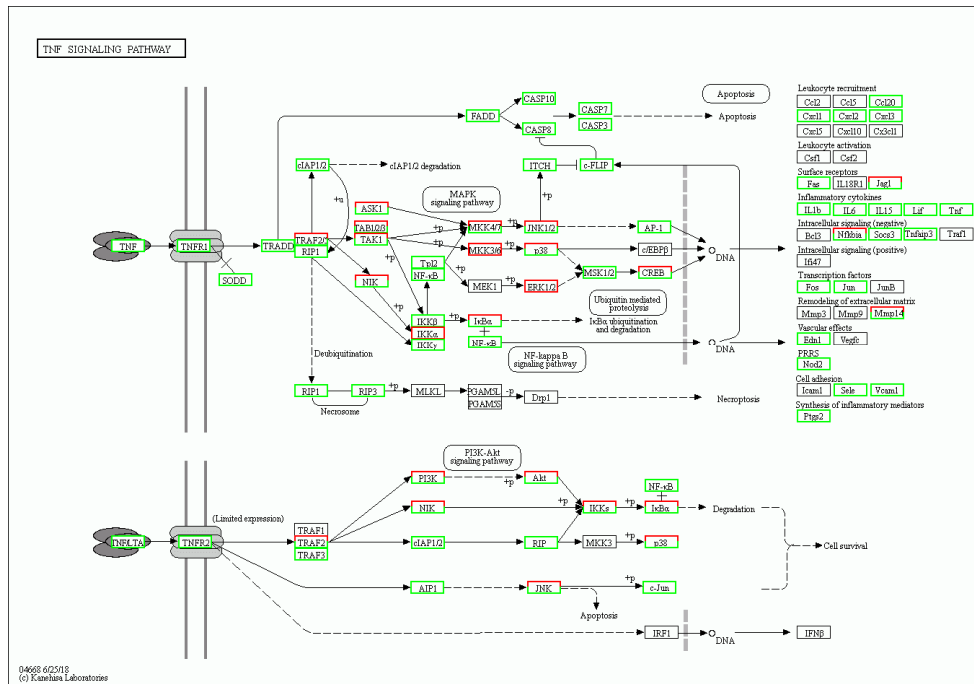







(A)

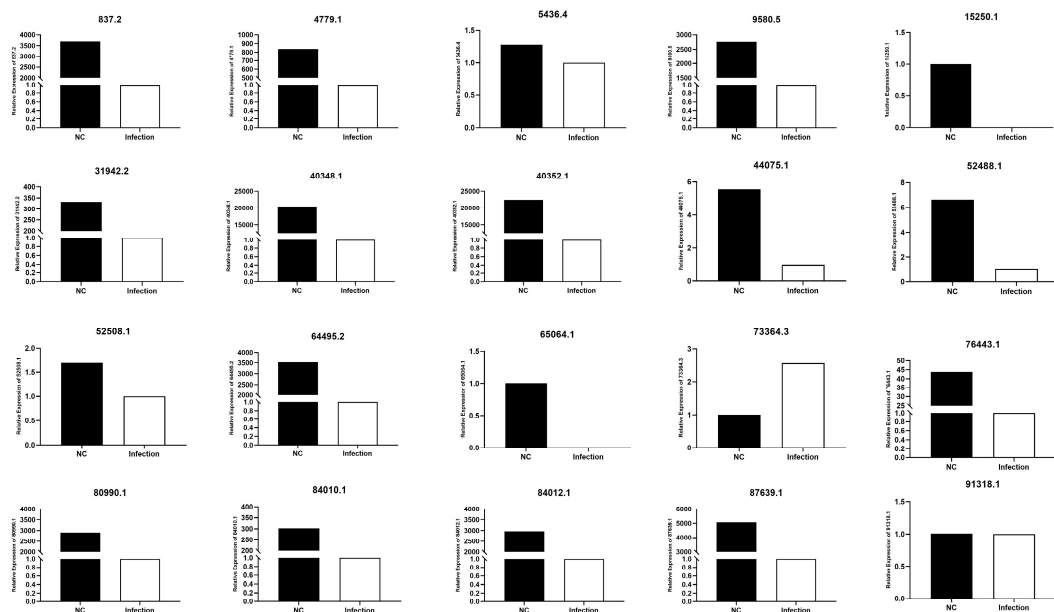

(B)

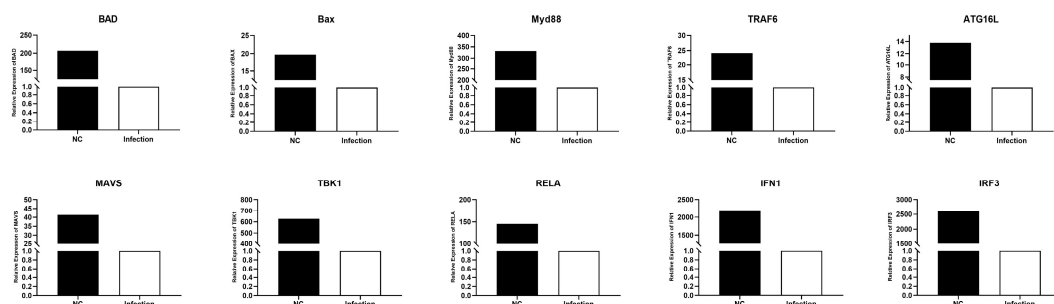

**Supplementary Figure S2.** RT-qPCR results of randomly selected 10 DEmRNAs and 20 lncRNAs.

Supplement: Supplementary file 1 [file viruses-14-02555-s001.zip › Supplementary Figures S1 and S2.pdf]
